# Early Epidural Blood Patch to Treat Intracranial Hypotension after Iatrogenic Cerebrospinal Fluid Leakage from Lumbar Tubular Microdiscectomy

**DOI:** 10.7759/cureus.3633

**Published:** 2018-11-26

**Authors:** Lukas Faltings, Kay O Kulason, Victor Du, Julia R Schneider, Shamik Chakraborty, Kevin Kwan, Bidyut Pramanik, John Boockvar

**Affiliations:** 1 Neurosurgery, Brain Tumor Center, Lenox Hill Hospital, Hofstra Northwell School of Medicine, New York, USA; 2 Neurosurgery, Zucker School of Medicine at Hofstra / Northwell, Hempstead, USA; 3 Radiology, Lenox Hill Hospital, Hofstra Northwell School of Medicine, New York, USA

**Keywords:** early epidural blood patch, tubular microdiscectomy, cerebrospinal fluid leakage

## Abstract

Management of cerebrospinal fluid (CSF) leak during minimally invasive lumbar tubular microdiscectomy poses challenges unique to the surgical approach. Primary repair can be limited via tubular retractor systems, and onlay graft and dural sealant are often the treatment of choice intraoperatively. Postoperative persistent CSF leak may lead to intracranial hypotension (IH) and positional headaches. Early epidural blood patch (EBP) efficacy in the treatment of spinal CSF leaks of both spontaneous and iatrogenic origin is well-established in numerous studies. However, there is no consensus on treatment of persistent IH symptoms for patients undergoing lumbar tubular microdiscectomy. We describe the clinical courses of two patients who were treated with early EBP for IH symptoms following CSF leak during tubular microdiscectomy. Both patients underwent intraoperative repair with onlay autologous tissue graft followed by dural sealant after discectomy was completed without evidence of pseudomeningocele, but they developed postoperative positional headaches and presumed IH. Both patients received an early EBP with an immediate and complete resolution of positional headaches sparing them reoperation and/or lumbar drainage. EBP should be considered as a first-line treatment to treat postoperative IH symptoms without pseudomeningocele after iatrogenic CSF leak during tubular microdiscectomy.

## Introduction

Tubular microdiscectomy has become a widespread method of treating lumbar disc herniation due to its minimally invasive approach and favorable benefit and risk profile [1]. A rare but challenging risk of this procedure is intraoperative durotomy, which postoperatively may lead to persistent cerebrospinal fluid (CSF) leakage, intracranial hypotension (IH), and positional headaches [2,3]. There is a lack of consensus regarding the optimal management of positional headaches after iatrogenic CSF leaks that occur during tubular microdiscectomy. Primary closure of a durotomy through the tubular retractor is often limited, so autologous tissue graft and dural sealants are necessary during the procedure if a CSF leak is encountered. However, if a patient continues to have postoperative positional headaches, reoperation may be needed to explore the wound and repair and reinforce the dural opening. Another potential treatment in this situation is lumbar drainage, which may facilitate closure of the CSF leak but necessitates an extension of the patient’s hospitalization in a monitored unit. Intralaminar epidural blood patches (EBPs) can also repair CSF leak and reverse IH of both spontaneous and iatrogenic etiologies [2,4,5]. An EBP consists of the injection of autologous blood directly into the lumbar epidural space; injected blood forms a seal at the leak site [6]. In this report, we describe two cases of tubular microdiscectomy-related CSF leak managed by epidural blood patches alone. The objective of this case series is to define the role of early EBP in the treatment of postoperative symptomatic IH resulting from intraoperative CSF leak associated with tubular microdiscectomy.

## Case presentation

Case 1

**Figure 1:**
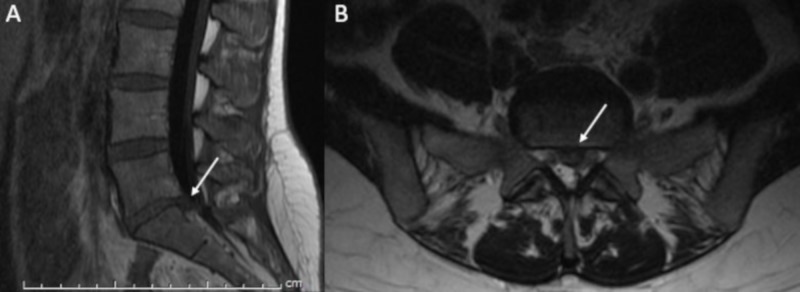
Case 1 diagnostic images. (A) Sagittal T1 FLAIR and (B) axial T2 weighted images of the lumbar spine demonstrate a left paracentral disk herniation with extruded disk compressing the left S1 nerve root. Arrows highlight areas of interest.

A 34-year-old female presented with intractable left leg radiculopathy. She reported moderate pain in her lower back with pain and numbness radiating down the left S1 distribution. She had no motor weakness or altered bowel or bladder function. Magnetic resonance imaging (MRI) demonstrated L5-S1 disc herniation compressing the S1 nerve root (Figure 1). The patient underwent an L5-S1 tubular hemilaminectomy and microdiscectomy. An intraoperative CSF leak was repaired with onlay autologous fat graft and dural spray sealant. Postoperatively, she developed positional headaches attributable to the CSF leak. She was treated with two EBPs at the L5/S1 interlaminar space on postoperative day one and two. The headaches resolved, and on outpatient follow-up two weeks postoperatively, she continued to deny headaches, and reported a complete resolution of her radiculopathy and resumption of daily activities pain-free. After her two-week follow-up, she continues to deny headaches and does not complain of pain.

Case 2

**Figure 2:**
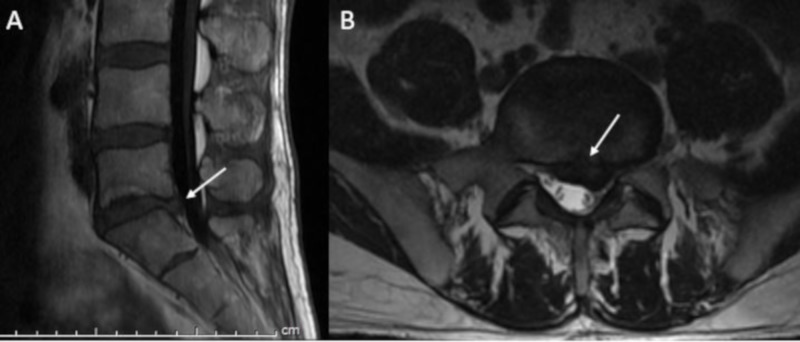
Case 2 diagnostic images. (A) Sagittal T1 FLAIR and (B) axial T2 weighted images of the lumbar spine demonstrate a large, central to left-sided disk herniation with extruded disk compressing the left S1 nerve root. Arrows highlight areas of interest.

A 49-year-old male presented with intractable and progressive pain in the lower back and left leg. Additionally, he had weakness of left plantar flexion. MRI revealed a large disc herniation at L5-S1 with compression of the left S1 nerve root (Figure 2). The patient subsequently underwent L5-S1 tubular hemilaminectomy and discectomy. An intraoperative CSF leak was repaired with onlay autologous fat graft and dural spray sealant. The patient developed positional headaches and received an EBP at the level of L5/S1 interlaminar space on postoperative day one with complete symptom relief. On outpatient follow-up two weeks postoperatively, he reported resolution of radiculopathy and denied headaches. Upon further follow-up, he continues to deny headaches and reports he is physically active.

## Discussion

The use of transmuscular tubular retractor systems represents a safe and efficacious surgical approach for the treatment of lumbar disc herniation [1,2,7]. CSF leaks are a frequent complication of spinal surgery with reported rates varying between 2 and 20% [8]. When iatrogenic CSF leak does occur in tubular microdiscectomy, unique challenges arise due to the limited surgical aperture provided by the retractor system. Management depends on comorbidities, difficulty of index procedure, and surgeon experience. A retrospective analysis determined that primary dural closure (±lumbar drain) successfully repaired intraoperative iatrogenic CSF leaks 73.4% of the time. In patients in whom primary closure was not achieved, a lumbar drain was placed and only 39.5% required re-exploration. However, those who required re-exploration had a statistically significant increase in length of stay (19.6 vs. 7.8 days), hospital admissions (2.1 vs 1.0), and infections (15 vs. 0). Authors concluded that the best management of a CSF leak is intraoperatively [8].

In our cases, the CSF leaks were intraoperatively sealed with autologous tissue and a dural sealant. However, both patients reported significant spinal headaches postoperatively indicative of IH. Although the precise diagnosis of IH requires a brain or spinal MRI, orthostatic headache is a classical symptom of IH [9]. If imaging is not conducted, IH is diagnosed with the presence of low opening pressure, spinal meningeal diverticulum, and symptom improvement after EBP [10], which were all present in both our cases. Additionally, there were no signs of pseudomeningocele in either patient, whose incisions were intact, dry, and flat. These clinical circumstances suggested their symptomatic IH could be definitively repaired by EBP without the need for more aggressive intervention such as lumbar drain or surgical re-exploration.

There are several complications associated with an EBP. For instance, paresthesia, neck ache, facial nerve palsy, and lumbovertebral syndrome have been reported after the procedure [11,12]. Additionally, seizure, severe headache secondary to pneumocephalus, and transient bradycardia have been reported during and immediately after the procedure [11,13-15]. Therefore, while re-imaging is not required to diagnose IH, it may be beneficial in future cases that are more ambiguous in order to avoid unnecessary surgical procedures. Nevertheless, EBP is a well-established and efficacious treatment for symptomatic IH with a high success rate [9].

We present our cases to outline a paradigm of using EBP as management for postoperative IH symptoms in patients with iatrogenic CSF leak associated with tubular lumbar microdiscectomy and without pseudomeningocele. EBP is a well-documented treatment for CSF leaks of both spontaneous and iatrogenic etiologies. In a study of 30 patients with spontaneous CSF leak, a complete cure was obtained in 77% of patients after application of EBP [16]. In another study, EBP completely resolved postural headaches resulting from a lumbar puncture in 90% of patients, and the use of a second EBP effectively treated the remaining 10% [17].

## Conclusions

We have demonstrated that the efficacy of EBP extends to the treatment of the postoperative sequelae of CSF leak associated with tubular microdiscectomy. In the absence of other complications such as pseudomeningocele, the application of EBP complements well the minimally invasive methodology of tubular spinal surgery, which may obviate the need for more aggressive treatments for CSF leak such as lumbar drain and exploratory reoperation. Patients may benefit from future studies examining the success rate of intraoperative EBP, which may enable patients to avoid re-exploration, and consequently an extended hospital stay and increased risk of infection.
